# Transcriptional response to VZV infection is modulated by RNA polymerase III in lung epithelial cell lines

**DOI:** 10.3389/fcimb.2022.943587

**Published:** 2022-07-25

**Authors:** Brianna M. Doratt, Elizabeth Vance, Delphine C. Malherbe, Mark T.W. Ebbert, Ilhem Messaoudi

**Affiliations:** ^1^ Department of Microbiology, Immunology and Molecular Genetics, College of Medicine, University of Kentucky, Lexington, KY, United States; ^2^ Sanders-Brown Center on Aging, University of Kentucky, Lexington, KY, United States; ^3^ Department of Internal Medicine, Division of Biomedical Informatics, University of Kentucky, Lexington, KY, United States; ^4^ Department of Neuroscience, University of Kentucky, Lexington, KY, United States

**Keywords:** RNA sensors, RNase L, VZV, antiviral innate immunity, transcriptome, RNA Polymerase III, MAVS, PKR

## Abstract

Ancestral RNA polymerase III (Pol III) is a multi-subunit polymerase responsible for transcription of short non-coding RNA, such as double-stranded short interspersed nuclear elements (SINEs). Although SINE ncRNAs are generally transcriptionally repressed, they can be induced in response to viral infections and can stimulate immune signaling pathways. Indeed, mutations in RNA Pol III have been associated with poor antiviral interferon response following infection with varicella zoster virus (VZV). In this study, we probed the role of Pol III transcripts in the detection and initial immune response to VZV by characterizing the transcriptional response following VZV infection of wild type A549 lung epithelial cells as well as A549 cells lacking specific RNA sensors MAVS and TLR3, or interferon-stimulated genes RNase L and PKR in presence or absence of functional RNA Pol III. Multiple components of the antiviral sensing and interferon signaling pathways were involved in restricting VZV replication in lung epithelial cells thus suggesting an innate defense system with built-in redundancy. In addition, RNA Pol III silencing altered the antiviral transcriptional program indicating that it plays an essential role in the sensing of VZV infection.

## Introduction

RNA polymerase III (Pol III) is a multi-subunit polymerase primarily responsible for the synthesis of essential housekeeping small noncoding RNAs (ncRNAs) (Paule and White, 2000). RNA Pol III is composed of 17 subunits including a central ten-subunit core containing the catalytic site; and uses specific promoter elements (type 1 [A- and C-Box], type 2 [A- and B-Box], type 3 [TATA-Box, proximal and distal elements]) and transcription factors (TFIIIA, TFIIIB, TFIIIC) (Ramsay et al., 2020; Lata et al., 2021). Pol III is found both in the nucleus and in the cytoplasm of eukaryotic cells where it has distinct functions (Chiu et al., 2009; Ramsay et al., 2020; Kessler and Maraia, 2021). Nuclear RNA Pol III is involved in the transcription of 5S ribosomal RNAs, transfer RNAs, U6 small nuclear RNAs and microRNAs (Carter-Timofte et al., 2019). In contrast, cytosolic RNA Pol III is involved in innate immune functions (Ramanathan et al., 2020; Lata et al., 2021). Indeed, mutations in POLR3A, POLR3C, POLR3E and POLR3F have been associated with increased susceptibility to viral and bacterial infections (Ramanathan et al., 2020; Lata et al., 2021). The structure of human RNA Pol III was recently mapped at high resolution and revealed that these mutations are localized in close proximity to DNA-binding regions (Ramsay et al., 2020).

RNA Pol III mediates transcription of transposons, which are ancient invasive elements that have amplified *via* retro-transposition and collectively comprise nearly half of the mammalian genome mass (Chen and Yang, 2017). Among transposons, short interspersed nuclear elements (SINEs) generate exclusively double-stranded ncRNAs, which are 75-500bp in length (Dunker et al., 2017). SINE ncRNAs are located both in the nucleus and in the cytoplasm. Depending on their cellular location, SINE ncRNAs affect distinct cellular processes with cytosolic Alu SINE interacting with protein kinase R (PKR) and modulating its activity while nuclear Alu SINE is a negative regulator of gene expression (Dunker et al., 2017). It is thought that cytosolic SINE ncRNAs can stimulate immune signaling pathways because they are not capped and thereby mimic immune activating pathogen associated molecular patterns (PAMP) (Burke and Sullivan, 2017). Indeed, Herpes simplex virus 1 (HSV-1) was shown to activate RNA Pol III transcription of human SINE loci (Panning and Smiley, 1994). Moreover, ALU SINE ncRNA expression is induced during infection with DNA viruses, including members of the adeno-, polyoma-, parvo-, and herpesviridae (Singh et al., 1985; Jang and Latchman, 1989; Panning and Smiley, 1993; Panning and Smiley, 1994; Williams, 1999; Karijolich et al., 2015).

Detection of viral dsRNA by the RIG-I pathway leads to the production of type I and type III interferons (IFNs) and induction of IFN-stimulated genes (ISGs) with antiviral activities (Platanias, 2005; Lopusna et al., 2013). In parallel, dsRNA is also sensed by oligoadenylate synthetases (OASs), which synthesize 2′-5′-linked oligoadenylates (2-5A) (Li et al., 2016; Whelan et al., 2019), leading to activation of latent endoribonuclease RNase L resulting in degradation of viral and host ssRNA (Dong and Silverman, 1995). Finally, dsRNA sensing by protein kinase R (PKR) results in protein synthesis shutdown and restriction of viral replication (Sadler and Williams, 2008). While RNase L and PKR antiviral activities are not dependent on IFN production (Whelan et al., 2019), the genes encoding OASs and PKR are ISGs; therefore, these pathways can be activated or reinforced by IFN production. Induction of SINE ncRNAs during viral infections can lead to the activation of these anti-viral pathways because they contain double stranded regions and can contain 5’ triphosphate ends (Dunker et al., 2017), which represent a virus-like immune activating PAMP (Burke and Sullivan, 2017) able to activate NF-KB in a manner partially dependent on the presence of the mitochondrial antiviral-signaling protein (MAVS), which functions as an adapter for the RIG-I-like receptors (Dorman et al., 1999). In addition, cytoplasmic ALU SINE ncRNA can form stable complexes with PKR (Chu et al., 1998; Williams, 1999). Thus, these ncRNAs have the potential to be recognized by cellular dsRNA sensors and could therefore serve as inducible immune signaling molecules.

Recent studies have shown that missense mutations in Pol III are associated with poor type I interferon production following varicella zoster virus (VZV) infection or reactivation and complications in the respiratory and central nervous systems (Ogunjimi et al., 2017). Pol III mutations were also detected in patients with recurrent VZV reactivation in the central nervous system indicative of impaired immunological sensing (Johnson et al., 2015; Warren-gash et al., 2017). In addition, cells from patients with Pol III deficiencies also demonstrated enhanced susceptibility to VZV infection *in vitro* (Ogunjimi et al., 2017; Carter-Timofte et al., 2019). These findings collectively suggest an essential role for Pol III in the antiviral response to VZV infection. Cytosolic RNA Pol III preferably converts poly(dA-dT) DNA into uncapped RNA and inhibition of Pol III led to reduced production of IFN-β in HSV-1 infected cells (Chiu et al., 2009). This preference for AT-rich motifs by cytosolic Pol III is a significant feature as VZV has a double-stranded DNA genome with 54% AT overall content and several AT-rich regions with 70–80% AT content that are not found in most other human herpesviruses (Paludan et al., 2011) and these AT-rich viral sequences could potentially be sensed as PAMPs. Thus, in this study, we probed the viral-sensing role of Pol III in the detection and initial immune transcriptional response to VZV infection.

VZV initiates primary infection by infecting epithelial cells in the respiratory tract, and then spreads *via* the hematogenous and axonal route to establish lifelong latent reservoirs in sensory neurons (Zerboni et al., 2014). The viremic phase and initial stages of skin disease of VZV primary infection are counteracted by the innate immune system in particular Type I IFN (Arvin et al., 1986; Biron et al., 1989; Ku et al., 2004; Duncan and Hambleton, 2015). Therefore, here we characterized the acute innate immune response following VZV infection of wild type A549 lung epithelial cells as well as A549 cells lacking specific RNA sensor MAVS (downstream mediator of dsRNA sensor RIG-I) (Alexopoulou et al., 2001; Lester and Li, 2014; Wu and Hur, 2015), RNase L and PKR (interferon-stimulated genes) (Silverman, 2007; Dauber and Wolff, 2009) in presence or absence of BRF1, the RNA Pol III transcription initiation factor subunit of the TFIIIB transcription factor complex, which is required for Pol III activity.

## Materials and methods

### Cell lines

The human lung epithelial cell line A549 (wild-type), and A549 knockouts (KO) for Toll-like receptor 3 (TLR3-KO), mitochondrial antiviral signaling protein (MAVS-KO), RNA-dependent protein kinase R (PKR-KO), and RNase L (RL6-KO) cells were culture in F12K medium supplemented with 5% fetal bovine serum (FBS), 100 IU/mL penicillin and 100 mg/mL streptomycin at 37 °C in humidified atmosphere with 5% CO2 (Li et al., 2016; Geerling et al., 2022).

### VZV propagation

VZV strain VR-916 was obtained from the ATCC and was propagated in MRC-5 cells as previously described (Griffiths and Haas, 2014; Meyer et al., 2015) using minimum essential medium (MEM) supplemented with 10% FBS as well as antibiotics (Pen/Strep) and glutamine. VZV-infected MRC-5 cells were frozen in FBS with 10% DMSO, stored in liquid nitrogen (LN_2_), and assayed by plaque assay in MRC-5 cells. For *in vitro* infection of A549 cells, VZV-infected MRC-5 cells were thawed, washed, and sonicated. Virus titers of sonicated prep was established by standard plaque assays on MRC-5 cells. A sonicated virus preparation was used to minimize RNA contamination from MRC-5 infected VZV.

### siRNA transfection and VZV infection

All cell lines were transfected at 70% confluence with 20 pmol (final siRNA used/well) of Dharmacon SMARTpool Brf-1 siRNA and or siRNA Universal Negative Control (Sigma) in serum-free Opti-MEM^®^ medium, using Lipofectamine^®^ RNAiMAX (Invitrogen). In short, 10 µM stock solutions of BRF1 or control (Ctrl) siRNA were diluted in 50μl of serum-free Opti-MEM^®^ medium and complexed with 3μl of Lipofectamine diluted in 50μl of Opti-MEM^®^ medium. Complexes were incubated at room temperature for 5 minutes and 100ul of the siRNA-lipid complex was added to each well. Complexes were removed 14 hours after transfection and cells were infected with sonicated VZV stock at an MOI of 0.1 in serum-free F12K medium. This MOI was chosen to ensure a higher infection level of A549 cells at the harvest timepoint (48 hours post infection). After adsorption for 1 hour at 37 °C, fresh F12K medium supplemented with 5% FBS was added to each well. Plates were returned to the incubator and the presence of cytopathic effect was monitored daily. Cells were harvested 48 hours post infection (hpi), 72 hours after transfection.

### Evaluation of protein expression by western blot

The levels of the BRF1 protein were evaluated by western blotting. Cells were lysed in RIPA lysis buffer (50 mm Tris-HCl, pH 7.2, 150 mm NaCl, 5 mm EDTA, 1% [wt/vol] (Thermo Scientific)) containing Phosphatase Inhibitor Cocktail (Pierce), and then cleared by centrifugation (15,000×*g* for 10 min). The protein lysates were mixed with 5 μl of NuPAGE LDS Sample Buffer 4X (Invitrogen) as well as 1M Dithiothreitol (DTT; Sigma) and heated at 70°C for 10 minutes. Denatured samples were resolved on an ExpressPlus PAGE Bis-Tris 4-20% gel (GenScript) and transferred to a nitrocellulose (NC) membrane (Millipore). The membrane was blocked with 5% nonfat dry milk, washed in Tris-Buffered Saline with 0.1% Tween 20 buffer (TBST), and probed with primary antibodies against BRF1 (1:1000 dilution; Rabbit anti-BRF1 from Bethyl) or actin (control) (1:1000 dilution, Mouse anti-actin from Santa Cruz Biotechnology). After washing in TBST, the membrane was incubated with HRP-conjugated secondary antibody (Santa Cruz Biotechnology anti-mouse BP-HRP 1:5000; or BioLegend HRP anti-rabbit IgG 1:2000). For detection, enhanced chemiluminescence was carried out using the ECL Plus kit (Amersham Biosciences Corp). Protein band quantification was performed using the Image Lab Software v.6.0.1.

### RNA extraction

Total RNA was extracted from infected/non-infected siRNA transfected A549 cells by using QIAzol reagent (QIAGEN) according to the manufacturer’s instructions. Chloroform was added to samples homogenized in QIAzol and tubes were shaken vigorously and centrifuged, and the upper aqueous RNA phase was transferred into a new tube. Ethanol was added to the samples and RNA purification was conducted using the miRNeasy Mini Kit (QIAGEN). Total RNA was DNAse treated, and quality assessed using the 2100 Bioanalyzer.

### cDNA library preparation and sequencing

For each sample, 1ug of total RNA was used to generate libraries using NEBNext Ultra Directional RNA Library Prep Kit for Illumina (New England Biolabs, MA, USA), according to manufacturer’s protocol. Each library was indexed using a unique molecular barcode for multiplexing and sequenced on the Illumina NovaSeq6000 platform (Illumina, San Diego, CA), with 120 bases in paired-end mode. RNA sequencing was performed in 72 samples yielding an average of 32-million paired-end reads/sample, average mapping to human genome was ~85%.

### Bioinformatic analyses

For reproducibility, all source code, data, and a Singularity container (Kurtzer et al., 2017) are available on GitHub at https://github.com/MessaoudiLab/RNA-Pol_III.

The GRCh38 reference genome and corresponding GTF file were downloaded from Ensembl at http://ftp.ensembl.org/pub/release-106/fasta/homo_sapiens/dna/Homo_sapiens.GRCh38.dna.primary_assembly.fa.gz and http://ftp.ensembl.org/pub/release-104/gtf/homo_sapiens/Homo_sapiens.GRCh38.104.gtf.gz, respectively (accessed January 2022). Both genomes were prepared for alignment using STAR v.2.7.10a (Dobin et al., 2013), where an SA index of 14 was used to index the GRCh38 genome and an SA index of 7 was used to index the VZV genome to account for the smaller genome size.

*STAR –runThreadN 12 –runMode genomeGenerate –genomeDir <path_to_output_folder>*

*–genomeFastaFiles <path_to_genome> –sjdbGTFfile <path_to_annotation_GTF>*

*–sjdbOverhang 99*

*–genomeSAindexNbases <SA_index>*

For initial data quality control and processing, RNA-Seq reads were first quality filtered and trimmed using Trim Galore (Bioinformatics, Martin, 2011, Bioinformatics) where both low-quality ends (‘–quality 30’) and adapters were trimmed from reads:

*trim_galore –paired ${read1} ${read2} –clip_r1 13 –clip_r2 13 –three_prime_clip_r1 2*

*–three_prime_clip_r2 2 –quality 30 –length 50 –fastqc*

The trimmed reads were then sequentially aligned to both the GRCh38 and VZV reference genomes using STAR v.2.7.10a (Dobin et al., 2013); specifically, all reads for a given sample were first aligned to GRCh38, and remaining unmapped reads were then aligned to the VZV reference.

*STAR –runThreadN 12 –genomeDir <path_to_indexed_genome> –readFilesIn <trimmedRead1> <trimmedRead2> –outSamtype BAM SortedByCoordinate –sjdbGTFfile <path_to_annotation_GTF>*

Raw gene counts were produced using FeatureCounts v.2.0.2 (Liao et al., 2014).

*featureCounts -T 10 –countReadPairs -p -t CDS,exon,gene,transcript,start_codon,stop_codon,five_prime_utr,three_prime_utr -a <path_to_annotation_GTF> -o <output_file> <path_to_input_bam_file_list>*

In addition, TPM (transcripts per million) counts for each sample were calculated from the raw counts using a custom R script (see GitHub for source code).

Finally, we performed additional quality control measures and RNASeq differential expression analyses using DESeq2 v.1.34.0 (Love et al., 2014) and Tidyverse v.1.3.1 (Wickham et al., 2019), following recommended practices (Bioconductor DESeq2 Vignette; accessed April 2022). for the following group comparisons: (1) non-transfected samples infected with VZV vs. non-transfected samples not infected with VZV, (2) transfected samples infected with VZV vs. transfected samples not infected with VZV, (3) siBrf1 transfected samples infected with VZV vs. non-transfected samples infected with VZV. Each group comparison was performed on each of the five cell lines: wild type (A549), MAVS knock-out, PKR knock-out, RL knock-out, and TLR3 knock-out. Briefly, we performed the following steps for each group comparison:

Before running the differential expression analysis, we performed additional data quality control on the DESeq2 data object. We first executed a DESeq2 regularized log transformation, which reduces the variability between samples for genes with low counts and normalizes the counts with respect to library size. We then performed a principal component analysis (PCA) using the regularized log transformed counts. Thirteen sample replicates failed to cluster with the other samples in their group, and thus were eliminated from subsequent analyses. For further quality control, we filtered the raw DESeq2 data object counts to include only genes with more than a sum of five reads across all samples.

Following final quality control steps, we performed differential expression between the groups previously specified using the DESeq() function. Briefly, the steps executed by the DESeq2 method are (1) estimation of size factors, (2) estimation of dispersion, and (3) negative binomial GLM fitting and Wald statistics. We used the following statistical models, as appropriate (see source code for exact implementation): (1) ‘design = ~ Condition’, where ‘Condition’ indicates the VZV infection status, (2) ‘design = ~ Transfection * Condition’, where ‘Transfection’ denotes the transfection type (I.e. non-transfected, siControl transfected, or siBrf1 transfected), (3) ‘design = ~Transfection_Process + Actual_Transfection’, where ‘Transfection_Process’ indicates whether the sample underwent any kind of transfection procedure, even if the transfection was a control, and ‘Actual_Transfection’ represents whether the sample received the siBrf1 transfection. Specifically, TRUE = siBrf1 transfection and FALSE = siControl transfection (i.e., not actually transfected). We did not include interaction terms in the third model because of collinearity. Lastly, we generated a results table by extracting the log2 fold change, *P-*value, and Benjamini-Hochberg adjusted *P-*value for the desired comparison (Benjamini and Hochberg, 1995).

### Statistical analysis

Statistical analyses were performed with GraphPad Prism version 9.3.1. P values less than 0.05 were considered statistically significant.

## Results

### VZV-infected A549 lung cell lines lacking antiviral defense genes display distinct transcriptome characteristics

Since VZV is a respiratory pathogen that targets lung epithelial cells during primary infection, we chose the A549 human lung epithelial cell line as an *in vitro* model to define the early innate immune responses. To gain insight into the role of Pol III in the acute anti-viral response, transcriptional responses were evaluated in wild type A549 as well as those lacking key RNA sensors 48 hrs following VZV infection (**Figure S1A**). MAVS is an adaptor protein part of the RIG-I signaling pathway while PKR and RNase L (RL) are interferon-stimulated genes and TLR3 is a cytosolic receptor for double-stranded RNA.

**Figure 1:**
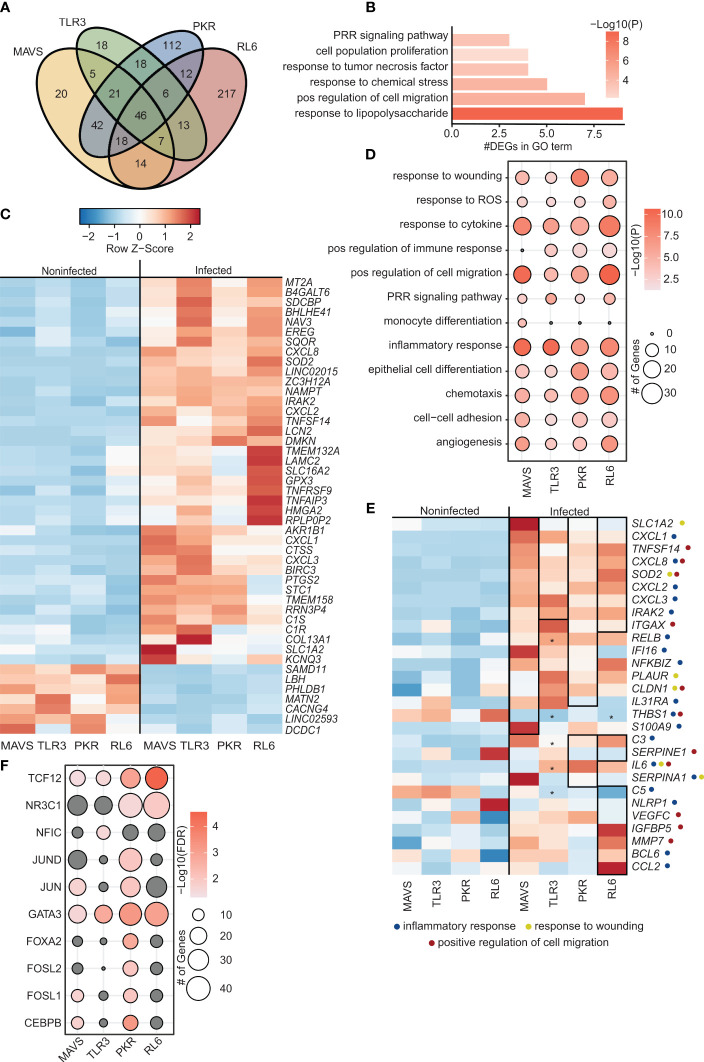
VZV-infected A549 lung cell lines lacking antiviral defense genes display distinct transcriptome characteristics. **(A)** Venn diagram of DEGs identified in siBRF1- VZV-infected cell lines relative to noninfected counterparts. **(B)** Bar graph depicting functional enrichment of the 46 DEGs common to all KO cell lines. Horizontal bars represent the number of DEGs enriching to each GO term. Colors are based on the GO terms with the lowest and highest –log(p-value) values. **(C)** Clustered heatmap comparing TPMs of the 46 DEGs common to all KO cell lines. Color range is based on scaled and centered TPM values. **(D)** Bubble plot representing functional enrichment of all DEGs in each KO cell line. Size of the bubble indicates the number of DEGs mapping to each GO term. Colors are based on the GO terms with the lowest and highest –log(p-value) values. **(E)** Clustered heatmap comparing TPMs of all DEGs in each KO cell line. Color range is based on scaled and centered TPM values. Box and * genes are differentially expressed in siBRF1- infected cell lines relative to noninfected counterparts. Symbol next to gene name represents GO terms the gene maps to. **(F)** Bubble plot representing transcription factors that are predicted to regulate the DEGs in each KO cell line. The size of the dot represents the number of genes, and the color represents –log(q-value) values. Analysis was performed using the Chea3 web browser.

Few differentially expressed genes (DEGs; 28) were detected in WT A549 cells (**Figure S1B**) and these genes played a role in interferon response, oxidative stress, lung function, cytoskeleton or are part of the long noncoding (lnc) RNA class (**Figure S1C**). In contrast, a more substantial transcriptional response was detected in MAVS-KO (173 DEGs), TLR3-KO (134 DEGs), PKR-KO (275 DEGs), and RL6-KO (333 DEGs) cells (**Figure S1B** and **Figure 1A**). In line with the magnitude of the transcriptional response, the number of viral reads was higher in the KO cell lines compared to WT, except for PKR (**Figure S1D**), indicating higher VZV replication. The majority of the DEGs, except for those detected in RL6, were upregulated with VZV infection. These DEGs showed modest overlap with a large number of unique DEGs detected in PKR-KO and RL6-KO (**Figure 1A**). The 46 common DEGs enriched to gene ontology (GO) terms associated with pathogen sensing and innate immunity such as “PRR signaling pathway”, “response to tumor necrosis factor”, and “response to lipopolysaccharide” (**Figure 1B**). Notable common upregulated DEGs played a role in complement activation (*LNC2, C1S, C1R*), cytokines and chemokines signaling (*CXCL1, CXCL3, CXCL8, TNRFSF9, PTGS2*), inflammation (*GPX3, PTGS2, EREG, ZC3H12A*), NF-KB pathway (*TNFAIP3, IRAK2*), interferon response (*MTA2*), wound healing (*LAMC2, COL13A1, SDCBP*), as well as stress and oxidative response (*EREG, NAMPT, GPX3*) (**Figure 1C**). In addition, some upregulated DEGs mapped to apoptosis and senescence (*BIRC3, SOD2, HMGA2, TMEM158*), the nervous system (*NAV3, SLC1A2, SLC16A2, KCNQ3, TMEM132A*) and the skin (*DMKN*) (**Figure 1C**). Furthermore, some upregulated genes have been associated with responses to herpesviruses (*TNFSF14*), RNA polymerase (*BHLHE41*) or are lncRNAs (*LINC02015*) (**Figure 1C**). Only 7 common DEGs were downregulated by VZV infection including SAMD11 (related to Polymerase II activity), *CACNG4* (chemokine), *LINC02593* (lncRNA) and *DCD1*, *PHLDB1*, *MATN2* (wound healing).

Analysis of all DEGs in the 4 KO cell lines (**Figure 1D**) showed enrichment to GO terms associated with inflammation (“inflammatory response”, “monocyte differentiation”, “positive regulation of immune response”) cell migration (“positive regulation of cell migration”, “chemotaxis”), signaling (“PRR signaling”, “response to cytokine”), and wound healing (“response to wounding”, “epithelial cell differentiation”, “angiogenesis”). Overall, VZV infection of cells lacking RNA sensors resulted in the upregulation of genes involved in innate immune response to pathogens including immune mediators (*CXCL8, S100A9, TNFSF14*), components of the NFKB signaling pathway (*IRAK2, RELB, NFKBIZ*), and interferon stimulated genes (I*FI16, IGFBP5*).

Specifically, in MAVS-KO cells, DEGs significantly upregulated by VZV infection were related to inflammation (*ITAGX, IL31RA, S100A9*), NF-KB activation (*RELB, NFKBIZ*), and wound healing (*PLAUR, CLDN1*). In addition, VZV upregulated expression of IFI16, an ISG important in the responses to herpesviruses (Dell’oste et al., 2015). In contrast, THBS1 (angiogenesis/integrin interaction) was significantly downregulated by VZV infection. In TLR3-KO cells, upregulated DEGs played a role in inflammation (*IL-6, RELB, C3*). As described for MAVS-KO cells, *THBS1* was significantly downregulated by VZV infection. Similarly, upregulated DEGs detected in PKR-KO cells also played a role in inflammation (*C3, ITGAX, IL-6, IL31RA, RELB, NFKBIZ*), antiviral innate immunity (*SERPINE1, IFI16*), wound healing (*PLAUR, CLDN1*) and lung function/disease (*IGFBP5*). VZV infection of RL6-KO cells led to the largest number of DEGs. However, these DEGs also played a role in antiviral innate immunity, (*SERPINE1, NLRP1, C3, ITGAX*), wound healing (*THBS1, VEGFC*), and lung function/diseases (*IGFBP5*) (**Figure 1E**).

We further evaluated the importance of each of the RNA sensors in the induction of the acute antiviral transcriptional program by identifying transcription factors that regulate the DEGs detected in response to VZV infection (**Figure 1F**). DEGs regulated by TCF12 and GATA3 were detected in all 4 cell lines. Only DEGs detected in PKR-KO cells were regulated by JUND, FOXA2 and FOSL2. On the other hand, only DEGs detected in MAVS-KO and PKR-KO were regulated by JUN and CEBP. Finally only DEGs detected in TLR3-KO are regulated by NFIC. Interestingly, although the largest number of DEGs was detected in RL6-KO cell line, these DEGs were primarily regulated by a handful of transcription factors (*GATA-3, NR3C1, TCF12*; **Figure 1F**). TCF12 and JUN transcription factors are associated with NF-KB. CEBP is involved in interferon responses. Notably, NFIC plays a role in Pol III initiation of transcription.

### Inhibition of polymerase III in A549 cells lacking antiviral defense genes leads to increased viral replication and altered transcriptomes

**Figure 2:**
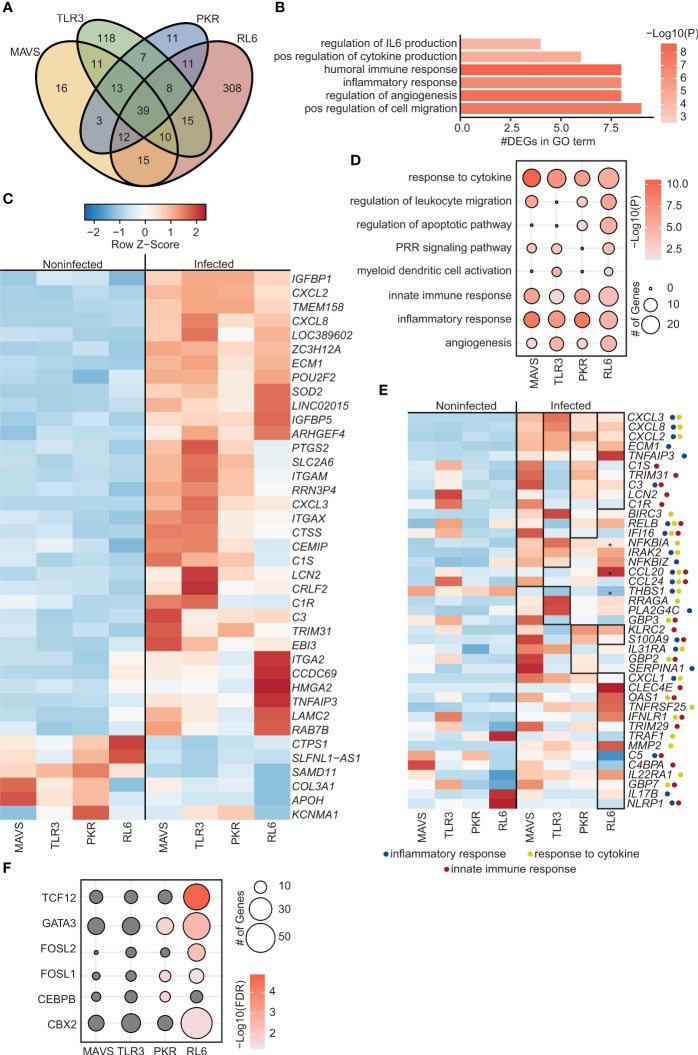
Inhibition of Polymerase III in A549 cells lacking antiviral defense genes leads to increased viral replication and altered transcriptomes. **(A)** Venn diagram of DEGs identified in siBRF1+ VZV-infected cell lines relative to noninfected counterparts. **(B)** Bar graph depicting functional enrichment of the 39 DEGs common to all KO cell lines. Horizontal bars represent the number of DEGs enriching to each GO term. Colors are based on the GO terms with the lowest and highest –log(p-value) values. **(C)** Clustered heatmap comparing TPMs of the 39 DEGs common to all KO cell lines. Color range is based on scaled and centered TPM values. **(D)** Bubble plot representing functional enrichment of all DEGs in each KO cell line. Size of the bubble indicates the number of DEGs mapping to each GO term. Colors are based on the GO terms with the lowest and highest –log(p-value) values. **(E)** Clustered heatmap comparing TPMs of all DEGs in each KO cell line. Color range is based on scaled and centered TPM values. Box and * genes are differentially expressed in siBRF1+ infected cell lines relative to noninfected counterparts. Symbol next to gene name represents GO terms the gene maps to. **(F)** Bubble plot representing transcription factors that are predicted to regulate the DEGs in each KO cell line. The size of the dot represents the number of genes, and the color represents –log(q-value) values. Analysis was performed using the Chea3 web browser.

To investigate the role of Pol III driven transcription in sensing of VZV, BRF1 was silenced by transient transfection of A549 cell lines with siRNA targeting BRF1. Silencing efficiency ranged from 94% reduction in WT cells to 71.5% reduction in MAVS-KO cells as assessed by determining BRF1 protein levels (**Figure S1E**). Loss of BRF1 expression led to a significant increase in VZV RL6-KO cells and modest increase (p<0.1) in WT and PKR-KO cell lines (**Figure S1D**). At the transcriptional level, BRF1 silencing led to a reduction in the number of DEGs induced by VZV infection in MAVS-KO (119 DEGs) and PKR-KO (104 DEGs) cell lines. In contrast, number of DEGs increased with BRF1 silencing in TLR3-KO (121 DEGs) and RL6-KO cells (418 DEGs) (**Figure S1B**, **Figure 2**). Most of the DEGs detected in MAVS-KO and PKR-KO cells were shared while most of the DEGs detected in TLR3-KO and RL6-KO were unique (**Figure 2A**).

A total of 39 common DEGs (6 downregulated and 33 upregulated DEGs) were detected (**Figure 2A**) that mapped to processes associated with response to infection including “inflammatory response”, “positive regulation of cytokine production” and “regulation of angiogenesis” (**Figure 2B**). Five downregulated DEGs were unique to BRF1 deficient cells including genes involved in lncRNAs (*SLFNL1-AS1*) and immune system (*CTSP1*), wound healing (*COL3A1*), and nervous system (*KCNMA1*) (**Figure 2C**). Of the 33 upregulated DEGs, 17 were unique to BRF1 knockdown cells (**Figures 1C**, **2C**). The uniquely upregulated DEGs in all BRF1 deficient cell lines were involved in complement (*C3, ITGAM, ITGAX*), immune system (*POU2F2, PTGS2, CRLF2*), interferon response (*IGFBP1, IGFBP5, TRIM31*), wound healing (*LAMC2, ITGA2, ECM1, CCDC69*), cell metabolism (*CEMIP, SLC2A6*), nervous system (*LAMC2*) and lncRNAs (*LOC389602*).

Additional analysis showed that DEGs detected across all 4 cell lines enriched to GO terms related to innate immune activation, cytokine signaling and inflammation (“PRR signaling pathway” “myeloid dendritic cell activation”, “innate immune response”, “response to cytokine”) (**Figure 2D**). Specifically, in MAVS-KO cells lacking BRF1, VZV infection led to significant upregulation of DEGs involved in complement (*C1R, C1S, C3, LNC2*), chemokines (*CCL20, CCL24*), NF-KB (*IRAK2, NFKBIA, NFKBIZ, TNPAIP3, RELB*), apoptosis (*BIRC3*), and antiviral immunity (*TRIM31, IFI16*) (**Figure 2E**). Similarly, VZV infection in TLR3-KO cells lacking BRF1 led to significant upregulation of genes important for complement and NF-KB activation (**Figure 2E**). Some unique DEGs detected in TLR3-KO include upregulation of phospholipase PLA2G4C and downregulation of THBS1 and antiviral genes GBP3. In PKR-KO cells deficient in BRF1, VZV infection significantly upregulated DEGs involved in inflammation including IL-17, TNFR genes and ISG (*OAS, GBP7, IFNLR1, TRIM29*) (**Figure 2E**). VZV infection of RL6-KO cells deficient in BRF1 led to a greater number of DEGs (**Figure S1B**). Some of the unique DEGs detected in this cell line include metalloprotease MMP7, transcription factor BCL6 and chemokine CCL2 while C5, NLRP1 and VEGFC were downregulated (**Figure 1E**). Additional analysis showed that DEGs detected in PKR-KO are regulated by GATA-3, FOSL1, and CEBPB while DEGs detected in RL6-KO are regulated by TCF12, GATA-3, FOSL1/2, and CBX2 (**Figure 2F**).

### BRF1 silencing differentially affects RNase L pathway

**Figure 3:**
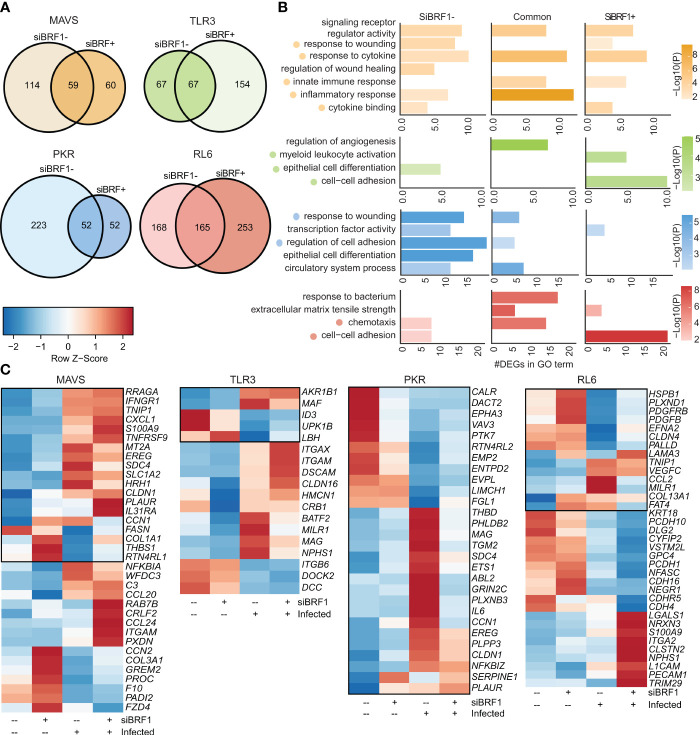
BRF1 silencing modulates antiviral transcriptional response. **(A)** Venn diagram of DEGs identified in siBRF1- infected vs noninfected cell lines relative to siBRF1+ counterparts. **(B)** Bar graph depicting functional enrichment of DEGs that are unique to siBRF1-, unique to siBRF1+, or common to all KO cell lines. Horizontal bars represent the number of DEGs enriching to each GO term. Colors are based on the GO terms with the lowest and highest –log(p-value) values. **(C)** Clustered heatmap comparing TPMs of DEGs mapping to the selected GO terms in **(A)**. Color range is based on scaled and centered TPM values. Boxed genes indicate differential expression in the siBRF1- condition.

Next, we directly compared transcriptional responses within each cell line in the presence and absence of BRF1 (**Figure 3A**). The lack of BRF1 had a significant impact on the acute transcriptional response to VZV infection with limited overlap between the two conditions. For instance, in the MAVS-KO cells, 173 DEGs were detected in non-silenced condition and 119 DEGs in silenced condition with 59 DEGs in common (**Figure 3A**). Despite this limited overlap, unique and common DEGs enriched to similar GO terms such as “response to cytokine”, “signaling receptor regulator activity”, and “cytokine binding” (**Figure 3B**). Only 4 DEGs were downregulated by VZV infection (*FASN, COL1A1, THBS1, RTN4RL1*) in BRF1-expressing MAVS-KO cells (**Figure 3C**). RTN4RL1 is involved in negative regulation of axon regeneration. COL1A1 and THBS1 are involved in wound healing. Upregulated DEGs in MAVS-KO cells expressing BRF1 encoded cytokines and chemokines (*CXCL1, TNFRSF9, IL31RA, CCL20, CCL24*), interferon response (*IFNGR1, MT2A*), inflammation (*S100A9, EREG, C3, NFKBIA*), and wound healing (*PLAUR, SDC4*). Downregulated DEGs were involved in immune responses (*PADI2*), lung function (*COL3A1*) and wound healing (*PROC, F10, CCN2*).

As described for MAVS-KO, BRF-1 silencing in PKR-KO cells led to a dramatic reduction in the number of differentially expressed genes with 275 DEGs in non-silenced condition and 104 DEGs in silenced condition, while 52 DEGs are shared (**Figure 3A**). While DEGs detected only in the presence of BRF1 enriched to GO terms such as “response to wounding” and “regulation of cell adhesion”, DEGs detected in the presence of BRF1 enriched primarily to “transcription factor activity” (**Figure 3B**). In the presence of BRF1, VZV infection induced the expression of several inflammatory genes (*IL6, SERPINE1, NFKBZ*) as well as those associated with wound healing (*PLAUR, CLDN1, ABL2, PHDB2, SDC4, THBD*). On the other hand, genes that play a role in signaling were downregulated (*VAV3, CLAR, PTK7*) (**Figure 3C**)

In contrast to MAVS and PKR-KO cells, BRF-1 silencing led to an increase in the number of DEGs in response to VZV in TLR3-KO cells with 134 DEGs in non-silenced condition, 221 DEGs in silenced condition, and 67 DEGs in common (**Figure 3A**). Unlike MAVS-KO, unique and common DEGs enriched to different GO terms with DEGs detected only in BFR1-expressing cells enriching to “epithelial cell differentiation”, while DEGs detected only after BRF1 silencing enriching to “cell-cell adhesion” and “myeloid leukocyte activation” (**Figure 3B**). In TLR3-KO cells expressing BRF1, VZV infection upregulated MAF (associated with Pol II) and downregulated ID3 (associated with NK-KB). In BRF1-silenced TLR3-KO cells, VZV infection upregulated DEGs involved in immune responses (*ITGAX, ITGAM, MILR1*), wound healing (*CLDN16, NPHS1*) and the nervous system (*DSCAM, MAG*). In addition, *BATF2* (associated with Pol II) was also upregulated. In contrast, *ITGB6* (virus receptor activity) and *DCC* (nervous system) were downregulated (**Figure 3C**).

BRF1 silencing in RL6-KO cells also led to an increased number of DEGs (418 vs 333) with 165 DEGs in common (**Figure 3A**). Despite the differential transcriptional response, enrichment was comparable with DEGs enriching to GO terms associated with chemotaxis “cell-cell adhesion” under both conditions (**Figure 3B**). In BRF1-expressing cells, upregulated DEGs mapped to cytokines (CCL2), innate immune responses (*MILR1*), NF-KB (*TNIP1*) and wound healing (*VEGFC, LAMA3, COL13A1, FAT4*) (**Figure 3C**). Downregulated DEGs also mapped to cytokines (*PDGFB*), NF-KB (*PDGFRB*), wound healing (PALLD), immune responses (CLDN4), stress (HSPB1) and nervous system (*EFNA2, PLXND1*). VZV infection of BRF1-silenced cells led to upregulation of DEGs involved in interferon responses (*TRIM29*), NF-KB (*LGALS1*), immune responses (*PECAM1*) and the nervous system (*NRXN3, CLSTN2, CDH4, L1CAM*). Downregulated DEGs in BRF1-silenced cells mapped mostly to the nervous system (*VSTM2L, NEGR1, PCDH1, NFASC*). Other downregulated DEGs were involved in apoptosis (*CYFIP2*), wound healing (*KRT18*) or affiliated with lncRNAs (*PCDH10-DT*).

## Discussion

Several studies have demonstrated a critical role for Pol III transcripts in modulating antiviral immune responses. For example, non-coding RNA moieties, such as SINE elements that are transcribed by Pol III, contain PAMPs and have been shown to be detected by viral RNA sensors; thus triggering activation of the NF-KB pathway (Karijolich et al., 2015). These Pol III transcripts are critical since clinical studies reported increased incidence of VZV reactivation and severity of primary infection in patients with Pol III mutations (Johnson et al., 2015; Warren-gash et al., 2017). Furthermore, these patients suffer from severe VZV infection but no other atypical infections, indicating a critical role for Pol III in the anti-VZV immune response. In this study, we sought to identify components of the viral sensing pathway that interact with Pol III transcripts during VZV infection. We compared transcriptional responses to VZV infection by WT A549 cells or those that were deficient in specific components of the RNA sensing machinery (MAVS, PKR, TLR3, RNase L) in the presence of absence of BRF1, an essential component of Pol III. MAVS is part of the RIG-I pathway, while PKR and RNase L are ISGs. Since herpesviruses can directly inhibit antiviral responses, we hypothesized that removal of these known components of the viral sensing pathway in A549 cells would results initially in a higher level of viral infection which would trigger heightened inflammatory and innate immune responses by the host. Indeed, herpesviruses encode tegument proteins that can inhibit these viral sensors. For example, HSV-1 encodes US11 which interferes with MAVS-RIG-I/MDA-5 interaction (Xing et al., 2012). HSV-1 also encodes virion host shutoff (vhs) RNase, which can block PKR activation early in infection (Glaunsinger, 2015). In addition, HSV-1 inhibits RNase L activity by synthesizing 2-5A antagonists (Cayley et al., 1984).

Compared to WT cells, VZV infection of KO cells led to increased detection of viral transcripts except for PKR-KO. In line with increased viral transcripts, VZV infection led to higher number of DEGs in all cell lines compared to WT cells. Notably, a large portion of these DEGs were involved in responses to pathogens, including immune responses, inflammation, and wound healing. In all KO cell lines, VZV infection led to upregulation of inflammatory responses (*GPX3, PTGS2, EREG, ZC3H12A*) and NF-KB related genes (*TNFAIP3, IRAK2*), as well as stress and oxidative responses (*EREG, NAMPT, GPX3*). In addition, VZV infection upregulated multiple genes involved in immune responses including chemokines (*CXCL1, CXCL2, CXCL3, CXCL8*), innate immune response and complement (*SDCBP, LNC2, C1S, C1R*), as well as wound healing (*LAMC2, COL13A1, SDCBP*). Two genes involved in anti-viral responses to herpesviruses were also significantly upregulated by VZV infection: TNFS14 in all cell lines and IFI16 in MAVS-KO and PKR-KO cells only. TNFS14 could function as a deterrent to infection by herpesviruses (Steinberg et al., 2011). Mutations in IFI16 lead to increased severity of Herpes Simplex virus infection (Dell’oste et al., 2015). In addition, among IFI16 related pathways are cytosolic sensors of pathogen-associated DNA and innate immune system. Interestingly, only infected PKR-KO cells upregulated *SERPINE1*, a component of antiviral innate immunity to influenza infection (Dittmann et al., 2015). It is therefore possible that the upregulation of *SERPINE1*, *TNFS14* and IFI16 could explain why VZV replication did not increase in PKR-KO cells compared to WT cells. Furthermore, many of the DEGs detected in PKR-KO cells after VZV infection were regulated by a number of transcription factors involved in immune and inflammatory responses (*CEBPB, NR3C1, FOSL2, GATA3*), NF-KB pathway (*TCF12, JUN*), damage response (*FOSL1*) and apoptosis (*JUND*).

In RL6-KO, C5 and NRLP1 were downregulated. C5 is a key member of the complement cascade and plays a major role in chemotaxis and inflammatory responses. NRLP1 is a mediator of apoptosis, and inhibition of apoptosis is a mechanism previously shown to increase virus production (Gerada et al., 2020). Downregulation of these genes may lead to increased viral replication in RL6-KO cells. The increased VZV replication in TLR3 deficient cells was initially unexpected as TLR3 is a cytosolic sensor of dsRNA. However, previous studies revealed that individuals with defects in genes of the TLR3 pathway suffer from severe primary VZV infection resulting in VZV encephalitis (Sironi et al., 2017). As TLR3 is expressed in human neurons and peripheral nerve Schwann cells (Prehaud et al., 2005; Goethals et al., 2010), TLR3 may play a pivotal role in controlling VZV spread in the nervous system.

Pol III inhibition by silencing of BRF1 subunit resulted in significantly higher VZV transcripts in RL6-KO cell line, and modest increases in WT and PKR-KO cells compared to the non-silenced RL6-KO and to the WT silenced cells. Furthermore, the effect of Pol III inhibition on the transcriptional response to VZV infection was dependent on the targeted RNA sensing component with decreased number of DEGs in MAVS-KO and PKR-KO and increase of DEGs in RL6-KO and TLR3-KO compared to their BRF1-expressing counterparts, thus suggesting a Pol III-dependent activation in MAVS-KO and PKR-KO cells. Notably, while BRF1 silencing was associated with an overall increased numbers of DEGs in the TLR3-KO cell line, it is noteworthy that several DEGs involved in innate immune responses (*LCN2, C1S, C1R, C3*), NF-KB pathway (*RELB*), interferon response (*GPB3*), responses to herpesviruses (*IFI16*) and in MAVS pathway (*TRIM31* that promotes the aggregation and activation of MAVS during viral infection (Liu et al., 2017)) were uniquely downregulated in TLR3-KO cells deficient in BRF-1 and thereby Pol III activity.

In BRF1 deficient cells, VZV infection of ISG knockout cell lines revealed a distinct activation pattern from the RIG-I sensor pathway. Indeed, interferon-related genes were significantly upregulated only in the ISG knockout cell lines with dramatic upregulation of CBX2-regualted genes (transcription factor regulating interferon gamma signaling), only in RL-6-KO cells. In addition, VZV also induced expression of genes involved in the response to herpesviruses (such as *TRIM31*) in PKR-KO and RL6-KO cells. RL6-KO cells deficient in BRF1 uniquely upregulated OAS, thus suggesting that this pathway can be engaged in the defense response against VZV infection in contrast to earlier studies showing that VZV infection did not lead to significant RNase L activation in MeWo cells (human skin fibroblasts) (Desloges et al., 2005). Furthermore, in absence of BRF1, the RNase L pathway appears to have a more prominent role in detecting and fighting VZV infection.

Finally, comparing the effect of VZV infection on BRF1-expressing and BRF1-silenced cells in the context of each RNA sensing pathway revealed different gene expression patterns showing the importance of Pol III in initiating anti-VZV response. Expression of genes involved in innate immune responses (chemokines, cytokines, NF-KB, wound healing) were modulated in both BRF1-expressing and BRF1-silenced MAVS-KO and RL6-KO cells, thus highlighting the redundancy built into responding to pathogens.

Interestingly, several DEGs detected in A549 lung epithelial cells following VZV infection were related to the nervous system. Transcription modulation of these genes was most notable in BRF1-expressing cells, especially the ISG knockout cell lines. However, since VZV establishes latent infection in sensory ganglia, modulating the expression of these genes may have an impact on the establishment of latency. In addition, this gene targeting may explain why the VZV-induced modulation of the transcriptome landscape of A549 lung cells was modest and could possibly be more pronounced in neurons.

**Figure 4:**
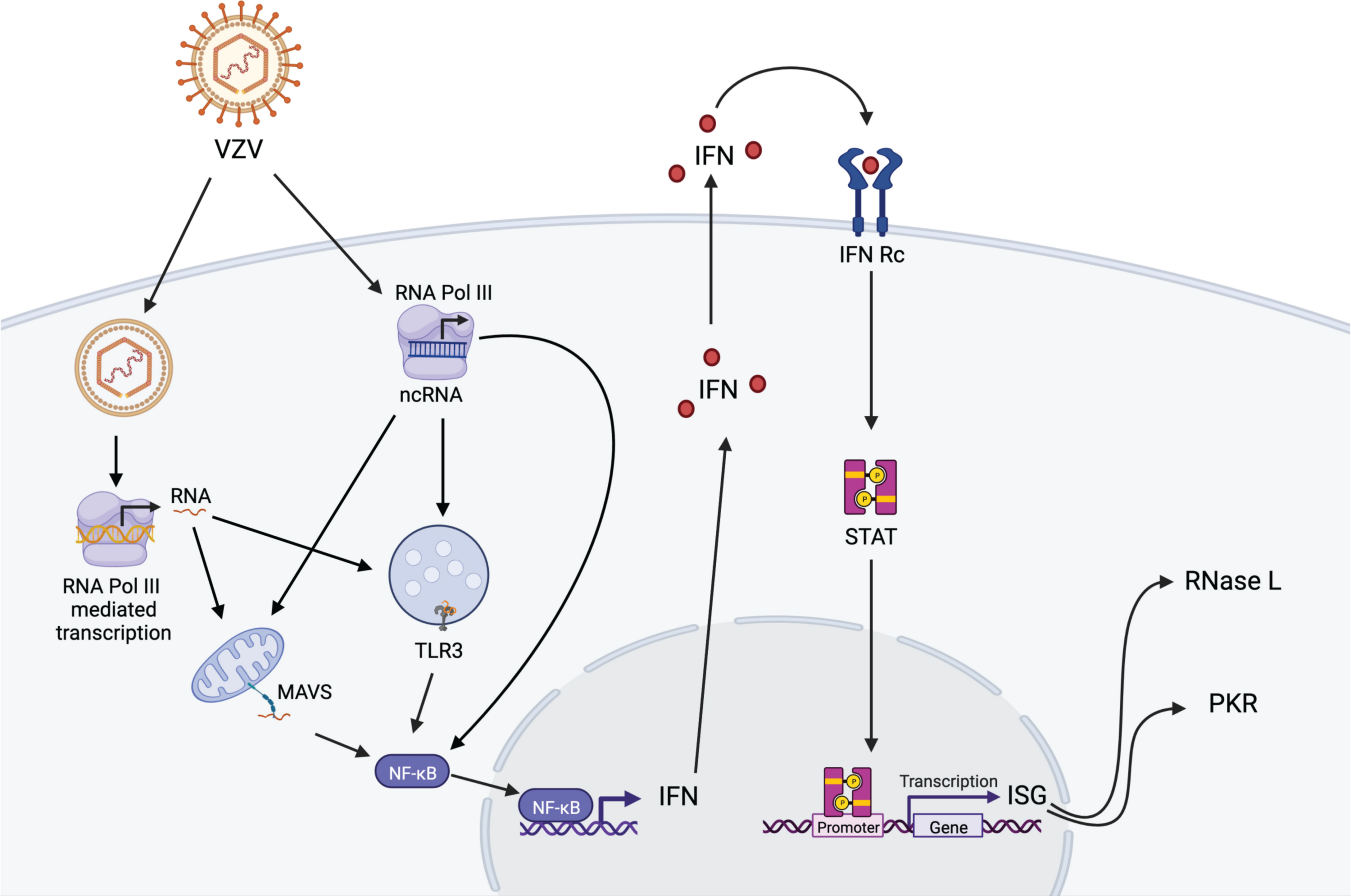
Proposed model of RNA Pol III-dependent activation of anti-VZV innate immune responses in lung epithelial cells. VZV DNA can be detected in the cytoplasm by cytosolic Pol III leading to activation of MAVS-dependent RIG-I pathway and TLR-3 pathway, which are also engaged by transcription of ncRNAs by cytosolic Pol III. Activation of these pathways results in translocation of NF-KB into the nucleus leading to interferon (IFN) response and activation of interferon-stimulated genes (ISG) RNase L and PKR in the initial host response against VZV infection. Figure generated on BioRender.com.

Overall, our data show that multiple components of the antiviral sensing and interferon signaling pathways are involved in restricting VZV replication in lung epithelial cells (**Figure 4**) thus suggesting a defense system with built-in redundancy. In addition, while silencing of each defense component led to a distinct transcriptome landscape upon VZV infection, RNase L deficiency led to a unique profile. Finally, our data suggest that RNA Pol III transcripts may act as immune sensors of VZV infection. Our study is not without limitations, notably the lack of specifically examining transcriptional changes within VZV-infected cells. Future studies could leverage the power of single cell RNA sequencing to address the impact within infected and bystander cells. Moreover, we were unable to measure the abundance of non-coding repetitive SINE because of the use of total RNA. Previous studies have shown that without separation of the nuclear and cytosolic compartments, the effect of SINE cannot be deciphered properly as its effects can be opposite of each other in the two compartments (Deininger, 2011; Karijolich et al., 2017). Future studies will focus on addressing the role of Pol III, and specifically SINE, in VZV sensing in critical target cells such as neurons and T cells.

## Data availability statement

The data presented in the study are deposited in the NCBI repository, accession number PRJNA850448.

## Author contributions

BD generated and analyzed data, performed bioinformatics analysis, and generated figures, EV performed bioinformatic analysis, DM analyzed data and wrote the initial draft, ME supervised bioinformatics analysis, and IM designed and supervised the study, edited the manuscript, and secured funding. All authors have read and approved the manuscript.

## Funding

This work was funded by NIH grant number R21AI143301.

## Acknowledgments

We thank Dr. Izabela Coimbra Ibraim for technical assistance. We thank Dr. Susan Weiss (University of Pennsylvania) for her generous gift of the A549 wildtype and knockout cell lines.

## Conflict of interest

The authors declare that the research was conducted in the absence of any commercial or financial relationships that could be construed as a potential conflict of interest.

## Publisher’s note

All claims expressed in this article are solely those of the authors and do not necessarily represent those of their affiliated organizations, or those of the publisher, the editors and the reviewers. Any product that may be evaluated in this article, or claim that may be made by its manufacturer, is not guaranteed or endorsed by the publisher.

## Supplementary material

Supplementary Figure 1 Silencing of components of antiviral defense affects viral replication and DEGs. **(A)** Experimental design for the study created partially on Biorender.com. **(B)** Number of differentially expressed genes (DEG) detected following VZV infection in the presence and absence of Pol III component BRF1. **(C)** Heatmap depicting the 28 DEGs detected in untransfected WT A549 cells with VZV infection. Boxed genes are differentially expressed in siBRF1+ transfected cells relative to untransfected counterparts. **(D)** Relative abundance of VZV transcripts detected in untransfected and in siBRF1-transfected cell lines by RT-qPCR. *P* values determined by 1-way ANOVA with **P*<0.05, ***P*< 0.01 and ****P*<0.001. siBRF1+ vs siBRF1- condition: # P<0.1 for WT and PKR; and P<0.05 for RL6. **(E)** Protein levels of BRF1 determined by Western Blotting. Actin was used as a loading control.
