# Supplementary figures and images for: Transcriptional response to VZV infection is modulated by RNA polymerase III in lung epithelial cell lines
Source: Front Cell Infect Microbiol. 2022 Jul 25;12:943587. doi: 10.3389/fcimb.2022.943587 (PMC9359802; doi:10.3389/fcimb.2022.943587)

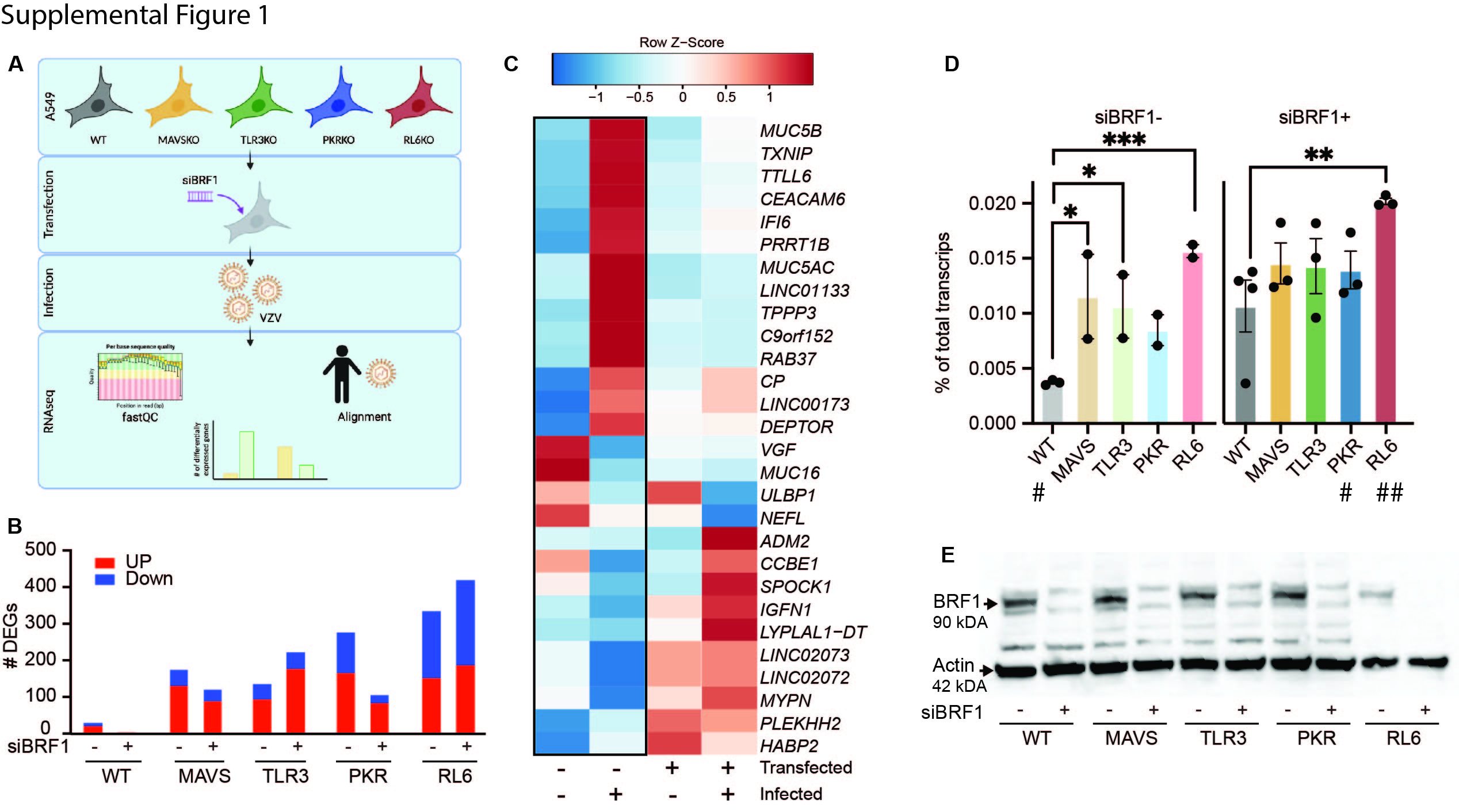

Supplement: Supplementary Figure 1 — Silencing of components of antiviral defense affects viral replication and DEGs. (A) Experimental design for the study created partially on Biorender.com. (B) Number of differentially expressed genes (DEG) detected following VZV infection in the presence and absence of Pol III component BRF1. (C) Heatmap depicting the 28 DEGs detected in untransfected WT A549 cells with VZV infection. Boxed genes are differentially expressed in siBRF1+ transfected cells relative to untransfected counterparts. (D) Relative abundance of VZV transcripts detected in untransfected and in siBRF1-transfected cell lines by RT-qPCR. P values determined by 1-way ANOVA with *P<0.05, **P< 0.01 and ***P<0.001. siBRF1+ vs siBRF1- condition: # P<0.1 for WT and PKR; and P<0.05 for RL6. (E) Protein levels of BRF1 determined by Western Blotting. Actin was used as a loading control. [file Image_1.jpeg]
